# MicroRNA circulating in the early aftermath of motor vehicle collision predict persistent pain development and suggest a role for microRNA in sex-specific pain differences

**DOI:** 10.1186/s12990-015-0069-3

**Published:** 2015-10-24

**Authors:** Sarah D. Linnstaedt, Margaret G. Walker, Joel S. Parker, Eunice Yeh, Robert L. Sons, Erin Zimny, Christopher Lewandowski, Phyllis L. Hendry, Kathia Damiron, Claire Pearson, Marc-Anthony Velilla, Brian J. O’Neil, Jeffrey Jones, Robert Swor, Robert Domeier, Scott Hammond, Samuel A. McLean

**Affiliations:** TRYUMPH Research Program, Chapel Hill, NC USA; Department of Anesthesiology, University of North Carolina, Medical School Wing C CB#7010, Chapel Hill, NC 27599-7010 USA; Department of Genetics, Lineberger Comprehensive Cancer Center, University of North Carolina, Chapel Hill, NC USA; Department of Cell Biology and Physiology, University of North Carolina, Chapel Hill, NC USA; Department of Emergency Medicine, Henry Ford Hospital, Detroit, MI USA; Department of Emergency Medicine, University of Florida College of Medicine-Jacksonville, Gainesville, FL USA; Department of Emergency Medicine, Albert Einstein Medical Center, Philadelphia, PA USA; Department of Emergency Medicine, Detroit Receiving, Detroit, MI USA; Department of Emergency Medicine, Sinai Grace, Detroit, MI USA; Department of Emergency Medicine, Wayne State University, Detroit, MI USA; The Cardiovascular Research Institute, School of Medicine, Wayne State University, Detroit, MI USA; Department of Emergency Medicine, Spectrum Health Butterworth Campus, Grand Rapids, MI USA; Department of Emergency Medicine, William Beaumont Hospital, Troy, MI USA; Department of Emergency Medicine, St Joseph Mercy Health System, Ypsilanti, MI USA; Department of Emergency Medicine, University of North Carolina, Chapel Hill, NC USA

**Keywords:** microRNA, Stress induced pain, Persistent axial pain, Motor vehicle collision, Sexual dimorphism, microRNA, Motor vehicle collision, African Americans, Musculoskeletal pain

## Abstract

**Background:**

Molecular mediators influencing the transition from acute to persistent musculoskeletal pain following common stress exposures such as motor vehicle collision (MVC) remain poorly understood. In this exploratory, proof of concept study, we compared circulating microRNA (miRNA) expression profiles in the early aftermath of MVC among individuals who did and did not subsequently develop persistent pain. Blood RNA samples were obtained from African American individuals (n = 53) who presented to the emergency department after MVC and were discharged to home after evaluation. The presence or absence of severe pain in the axial region, the most common and morbid region in which post-MVC pain occurs, was assessed 6 weeks following MVC via standardized questionnaire. miRNA expression was determined using miRNA-sequencing; nonparametric analyses were used to compare miRNA expression levels among individuals with and without persistent pain.

**Results:**

Thirty-two mature miRNA were differentially expressed (p < 0.05) in those with and without severe axial pain at 6 weeks. miR-135a-5p, a regulator of the serotonin receptor that is known to be stress-responsive, differed most significantly between groups (p = 3 × 10^−4^). This miRNA, and miR-3613-3p (p = 0.001) survived correction for multiple testing (FDR = 0.15) in this small sample. Interestingly, differentially expressed miRNA were enriched for X chromosome location. In secondary analyses, the eight X chromosome miRNA were (a) more significantly associated with axial pain in women than men, (b) expressed more highly in the peripheral blood of women than men, and (c) predicted in pathway analyses (DIANA miRPath v 2.0) to regulate neuronal and neuroendocrine pathways previously implicated in various pain pathologies.

**Conclusions:**

These results show that circulating miRNA predict persistent severe axial pain after MVC and suggest that they may be involved in the pathogenesis of post-traumatic musculoskeletal pain. However, further studies are needed to determine if these miRNA play a direct causal role.

**Electronic supplementary material:**

The online version of this article (doi:10.1186/s12990-015-0069-3) contains supplementary material, which is available to authorized users.

## Background

MicroRNA (miRNA) are small non-coding RNA molecules that regulate gene expression by binding target mRNA. During the past decade, the study of miRNA has transformed understanding of the regulation of major biological pathways [1, 2] and advanced understanding of the pathogenesis of a number of common diseases (e.g. [3–5]). Substantial evidence suggests that miRNA may play a critical role in molecular pathways underpinning diverse pain conditions (e.g. [6–8]). Most of this evidence comes from animal studies; further human studies are needed which prospectively evaluate the potential role of miRNA in persistent pain development after potential triggering events.

One common potential triggering event for persistent pain is exposure to a motor vehicle collision (MVC). More than fifty million MVCs occur each year worldwide [9], and more than 4 million individuals present to US emergency departments (ED) each year for care after MVC [10, 11]. Ninety percent of individuals presenting to US EDs for care after MVC are discharged home after evaluation with little or no identifiable tissue injury [12]. A substantial proportion of these individuals develop musculoskeletal pain, most commonly in the axial region (neck, shoulders, and/or back) [13].

The molecular mechanisms responsible for axial pain (AP) development after MVC remain poorly understood. This lack of understanding is a major barrier to the development of more effective preventive interventions. If miRNA play an important role in the pathogenesis of post-MVC AP, then studies identifying differences in miRNA populations among those who do and do not subsequently develop these outcomes may advance understanding of post-MVC AP pathogenesis [14–16]. Such studies must be feasible and ethical, and should obtain miRNA samples from tissue relevant to disease pathogenesis and/or be representative of such tissue.

Several lines of evidence suggest that blood is not only a feasible source of miRNA for studies of post-MVC musculoskeletal pain pathogenesis, but also that blood miRNA studies may provide pathogenic insights [17–19]. First, stress systems appear to be involved in the pathogenesis of persistent pain after MVC [20–23], and blood-borne stress and immune-related factors are an important component of the systemic stress response [24]. In addition, RNA expression patterns in blood, CNS, and endocrine tissues are strongly correlated [15, 25, 26], and a number of previous studies have linked miRNA expression in blood with neurologic disease outcomes [27–30]. Finally, despite challenges related to tissue-specific gene expression, the study of blood miRNA expression has led to important new understanding of diverse diseases, including pain conditions [27, 31, 32].

In this prospective study, we compared ED blood miRNA profiles among individuals who presented to the ED for evaluation after MVC. We hypothesized that ED blood miRNA profiles would differ among those who did and did not have severe persistent AP 6 weeks later.

## Results

### Cohort

Characteristics of the study sample (n = 53) are shown in Table 1. Samples were drawn from a large prospective cohort study of African Americans (R01AR060852); all participants were African American (AA) and nearly six in ten were female. Most were less than 40 years old, had some college education, made less than 40 K annually, and were overweight (average BMI = 30). All individuals in this study presented to the ED within 6 h of MVC and most arrived within 1 h. Only individuals who were discharged from the ED who reported no lacerations, avulsions, or major tissue injury were included. Additionally, participants all had a severity score of 1 on the Abbreviated Injury Scale (AIS) [33], indicating minimal anatomical injury. Six weeks following MVC, severe AP was present in 27/53 participants. Among these 27 individuals who developed severe AP, 16 (59 %) were women.

**Table 1:** Study characteristics

Characteristic	
Participants, n	53
Age, years, mean (SD)	37 (13)
Females, n (%)	31 (58)
Education, n (%)	
Some or all of high school	16 (30)
Some college	25 (47)
College	9 (17)
Post-college	2 (4)
Income, n (%)	
0–20 K	10 (19)
20–40 K	18 (34)
40–80 K	10 (19)
>80 K	2 (4)
Body mass index, mean (SD)	30 (6)
Time to ED presentation in minutes, median	54

### miRNA sequencing quality assessment

An average of 9 million sequencing reads were obtained per participant from blood samples obtained in the ED in the early aftermath of MVC. More than 95 % of these miRNA aligned with miRNA in miRBase, indicating that the majority of the sequencing reads were mature miRNAs (vs. degradation products, linker–linker contaminants, etc.). Mature miRNA with an average of ≥300 sequencing reads across all 53 samples (n = 376 miRNA) were included in analyses. Relative proportions of several miRNA typically found in peripheral blood were very similar to those reported previously [15, 34] (data not shown).

### Evaluation of ED miRNA expression levels among those who did and did not develop persistent AP 6 weeks following MVC

Thirty-two of 376 (9 %) miRNA detected in ED blood samples were differentially expressed at the p <0.05 level among those who did and did not report severe post-MVC AP at 6 weeks, with fold differences ranging from −3.71 to 2.49 (Table 2, sequencing read counts used for determination of mean fold differences for each miRNA are included in Additional file 1: Table S1). Nine of these 32 differentially expressed miRNA have previously been associated with pain and/or stress system physiology in neurological tissue and/or blood (see ‘Ref’ column, Table 2). Two of the 32 differentially expressed miRNA, miR-135a-5p (p = 3 × 10^−4^) and miR-3613-3p (p = 0.001), met our pre-hoc significance level threshold for multiple testing of 0.15, corresponding to a p value cut-off of <0.003 [35].

**Table 2:** microRNA in whole blood circulating in the early aftermath of motor vehicle collision in African Americans that predict axial pain development 6 weeks after MVC trauma

microRNA	Mean fold difference^a^	p value^b^	Previous assoc^c^
miR-135a-5p	2.49	*3* *×* *10* ^*−4*^	S, N [47, 48]
miR-3613-3p	2.02	*0.001*	
miR-19b-3p	1.67	0.004	S, P [49, 51]
miR-502-3p	1.75	0.004	
miR-500a-3p	1.39	0.005	
miR-1296-5p	1.99	0.006	S [50]
miR-454-5p	1.58	0.010	
miR-99a-5p	1.48	0.010	P [71]
miR-501-5p	−1.15	0.011	
miR-362-5p	1.41	0.013	
miR-154-5p	1.09	0.015	
Let-7a-3p	1.48	0.020	S, P [50, 72]
miR-185-5p	−3.45	0.021	P [52]
miR-339-5p	1.31	0.023	
miR-29c-5p	1.67	0.023	
miR-4659b-3p	−2.19	0.023	
miR-15b-5p	−1.22	0.026	
miR-329-3p	1.68	0.026	
miR-20b-5p	1.35	0.029	S [73]
miR-500b-5p	1.38	0.029	
Let-7f-2-3p	1.43	0.029	
miR-7-5p	−2.12	0.033	P [53, 73]
miR-378a	1.37	0.034	
miR-3130-5p	1.91	0.034	
miR-532-5p	1.31	0.036	
miR-345-5p	1.62	0.037	
miR-16-5p	−2.70	0.043	
miR-18a-3p	1.49	0.044	
miR-337-3p	−1.06	0.045	
miR-26b-3p	−3.71	0.046	P [54]
miR-26a-5p	−2.52	0.048	
miR-151b	1.33	0.048	

^a^Mean fold difference was calculated by dividing the average sequencing read counts for individuals developing axial pain by the average sequencing read counts for individuals who recover

^b^p values were calculated using the Mann–Whitney U test. Italicized miRNA remained significant after correcting for multiple testing (FDR = 0.15)

^c^Previous assoc = references describing a previously identified role for the miRNA in stress system biology (S), pain pathobiology (P), or neuropsychiatric disease (N)

### Validation of miRNA sequencing results using RT-qPCR

**Fig. 1:**
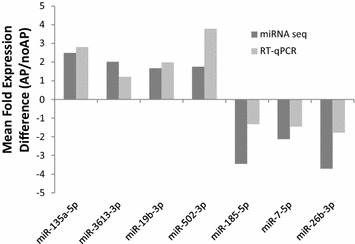
RT-qPCR validation of microRNA that predict axial pain (AP) development following motor vehicle collision (MVC). *Dark grey bars* represent expression differences calculated using mean microRNA sequencing counts in individuals developing AP divided by mean microRNA sequencing counts in individuals recovering after MVC. *Light grey bars* represent expression differences in the same two groups using mean cycle thresholds generated via RT-qPCR. Spearman Correlation assessing correlation of the two methods: r = 0.786, p = 0.036

Technical and qualitative validation of miRNA sequencing results was performed on a random subsample of 7 of the 32 differentially expressed miRNA using reverse transcription quantitative-PCR (RT-qPCR) [36]. In each case, concordance between miRNA sequencing results and RT-qPCR results was observed for direction of differential expression (i.e., positive or negative expression difference, Fig. 1). Magnitude of direction of effect was also generally similar. The Spearman Correlation between the two methods was also calculated: r = 0.786 p = 0.036.

### Differentially expressed miRNA were enriched for X chromosome location

**Fig. 2:**
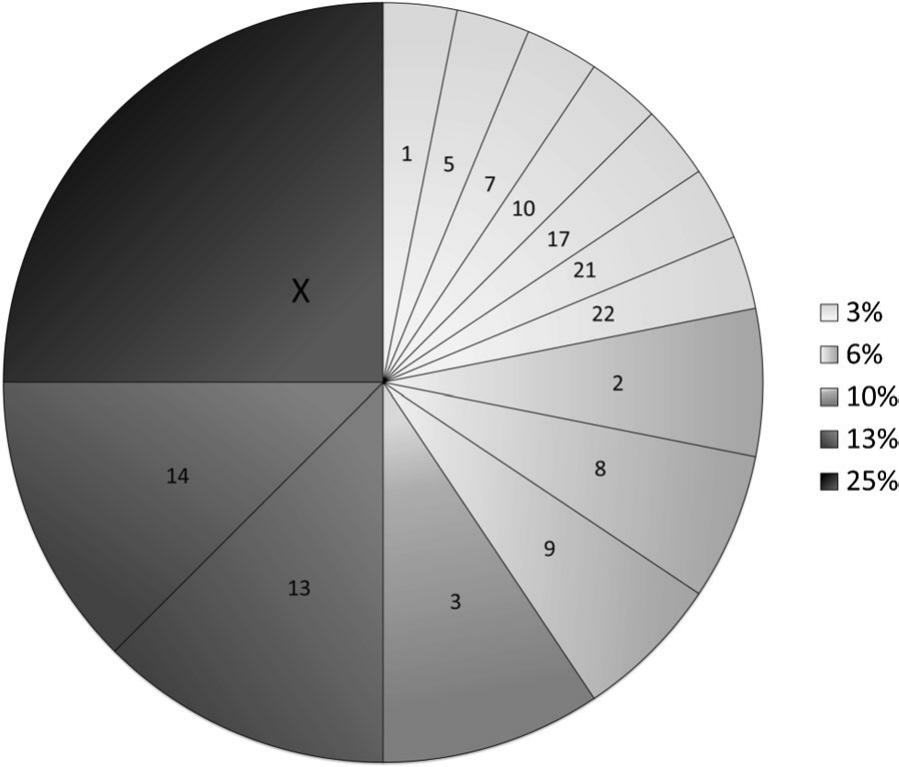
microRNA that predict axial pain (AP) development are transcribed from 14 different chromosomes. The percentage of miRNAs (out of the 32 associated with AP, Table 2) that originate from each of the 14 chromosomes are represented by pieces of the pie chart based on shading (*lightest shading* = 3 % and *darkest shading* = 25 %, with intermediate percentages having intermediate *shading colors*, see legend). The names/number of each chromosome are labeled inside each piece of the pie

Specific data regarding chromosomal origin and strand (sense or antisense) for the 32 differentially expressed miRNA is shown in Additional file 1: Table S1. The 32 miRNA predictive of severe post-MVC AP at 6 weeks were enriched for gene location on the X chromosome (Fig. 2) in comparison to all X chromosome miRNA identified in the sample (8/32 (25 %) vs. 28/376 (8 %), p = 0.038). This holds true despite the fact that approximately 8 % of the detected blood miRNA originate from the X chromosome.

### X chromosome miRNA identified in this study are more highly associated with the development of persistent AP following MVC in women than men

Because X chromosome gene expression can be sex-dependent [37], after we observed the X chromosome enrichment of differentially-expressed miRNA in our sample, we assessed for interactions between sex and the effect on severe persistent AP of differentially expressed X chromosome miRNA. Even in the relatively small samples of men and women assessed, sex × miRNA interactions were significant at the p <0.05 level for miR-362-5p and let-7f-2-3p, and were present at the trend level for miR-501-5p and miR-500b-5p (Table 3, Additional file 1: Table S1). Much greater differences in expression according to pain outcome were observed in women (n = 31) vs. men (n = 22) (Table 3). Seven of the 8 X chromosome miRNA were significantly associated with AP development in women, whereas none of the X chromosome miRNA were associated with AP development in men (Table 3). Additionally, after discovering significant sex × miRNA interactions for the X-chromosome miRNA described above, we assessed for additional interactions between participant sex and miRNA associated with persistent AP. A significant sex × miRNA interaction was present for two additional miRNA, miR-1296 (p = 0.017) and Let-7a-3p (p = 0.022) (see Additional file 1: Table S1 for results of all sex × miRNA interactions). These two miRNA are significantly associated with persistent severe AP in women (p < 0.001 and 0.001) but not in men (Additional file 2: Table S2).

**Table 3:** Association of Emergency Department expression levels of microRNA (miRNA) from the X chromosome with persistent axial pain following motor vehicle collision in women vs. men and assessment of interaction between sex of an individual and miRNA

miRNA	Women (n = 31)		Men (n = 22)		Interaction (sex × miRNA)
	Fold difference	P value^a^	Fold difference	P value^a^	P value^b^
miR-502-3p	2.17	*0.001*	1.17	0.562	0.400
miR-500a-3p	1.39	*0.009*	1.36	0.300	0.251
miR-501-5p	−1.41	*0.037*	1.45	0.217	0.071
miR-362-5p	1.32	*0.049*	1.55	0.151	*0.030*
miR-20b-5p	1.47	*0.012*	1.12	0.847	0.878
miR-500b-5p	2.60	*0.014*	−1.36	0.606	0.054
Let-7f-2-3p	6.9	*0.001*	−2.06	0.797	*0.015*
miR-532-5p	1.24	0.093	1.41	0.243	0.294

^a^p values were calculated using the Mann–Whitney U test

^b^p values for the interaction term were calculated using a logistic regression model adjusted for age and site. P values meeting a significance threshold of p <0.05 are italicized

### X chromosome miRNA identified in this study are expressed more highly in the blood in the early aftermath of MVC in women than men

Based on previous reports showing higher expression of some X chromosome genes in women than men (most notably in brain tissue [38] and possibly due to mechanisms such as escape from X chromosome inactivation [39]), we assessed whether the X chromosome miRNA identified in this study are expressed at higher levels in women than men. All eight miRNA were expressed at higher levels in women than in men developing severe AP, although only one was statistically significant (miR-502-3p, p = 0.017) (Table 4).

**Table 4:** Emergency Department expression level differences of microRNA (miRNA) from the X chromosome in women vs. men developing persistent axial pain following motor vehicle collision

miRNA	Expression difference^a^ (women/men)	P value
miR-502-3p	1.80	0.017
miR-500a-3p	1.43	0.112
miR-501-5p	1.38	0.209
miR-362-5p	1.08	0.773
miR-20b-5p	1.60	0.126
miR-500b-5p	1.37	0.417
Let-7f-2-3p	1.78	0.252
miR-532-5p	1.15	0.563

^a^Expression difference is the mean sequencing read counts of the specified miRNA in women who have axial pain at 6 weeks divided by the mean sequencing read counts of men who have axial pain at 6 weeks

### Evaluation of biologic pathways targeted by AP-associated X chromosome miRNA

Using DIANA miRPath v 2.0 [40], we assessed for molecular pathways (KEGG pathways [41]) overrepresented in predicted targeting by these 8 X chromosome miRNA. The pathways with the highest number of gene transcripts targeted by the eight X chromosome miRNA in Table 2 (i.e. most statistically significant enrichment) out of ~450 KEGG pathways that DIANA miRPath queries are shown in Table 5 (false discovery rates (FDRs) calculated via permutation testing [42]). These pathways include neuronal and neuroendocrine pathways such as the Long Term Potentiation pathway (p = 1.67 × 10^−9^, FDR ≤ 0.08), the Axon Guidance pathway (p = 1.32 × 10^−7^, FDR ≤ 0.22), Neurotrophin signaling (p = 7.35 × 10^−7^, FDR ≤ 0.22) and the Dopaminergic synapse signaling pathway (p = 1.56 × 10^−6^, FDR ≤ 0.33). Substantial evidence supports an important role for these pathways in mediating physiologic responses to stress and the pathogenesis of acute and persistent pain [43–45]. In addition to assessing X-linked miRNA targeted pathways, we also assessed which pathways might be enriched in targeting by all 32 miRNA associated with persistent AP development. This data is provided in Additional file 3: Table S3.

**Table 5:** DIANA miRPath predicted KEGG pathways enriched in targeting by X chromosome miRNA differentially regulated in the early aftermath of MVC trauma in AA individuals who develop AP following MVC vs. those who recover

KEGG pathway	P value	Example of predicted targets
Ubiquitin mediated proteolysis	2.15 × 10^−10^	
Long-term potentiation	1.67 × 10^−9^	PRKCA*, CAMK4, GRIA1, PPP3CC, KRAS, CALM2*, GRIA2, PPP3CA, GRM1, RPS6KA3*, EP300*, GNAQ*
Axon guidance	1.32 × 10^−7^	GSK3B*, ABLIM3, SEMA5A, EPHA5, ROCK2*, PAK7, ROBO2, SEMA3C*, SRGAP1*, PPP3CC, KRAS, EPHA7, PPP3CA, PTK2*, RASA1*, NFAT5*, EPHB4*, UNC5C, CFL2*, SEMA3D, SEMA3A*, PLXNC1*, EPHA4*, SEMA3E, UNC5D
ErbB signaling	7.04 × 10^−7^	
Neurotrophin signaling	7.35 × 10^−7^	GSK3B, NTRK2*, CAMK4, CRK, SORT1*, NTRK3*, FRS2*, MAP3K1*, KRAS, CALM2*, JUN, MAPK8, SOS1*, RPS6KA3*, GAB1, AKT3*, CAMK2B, FOXO3, MAP2K1, PRDM4, RAP1B, MAP3K5
Insulin signaling	8.72 × 10^−7^	
Dopaminergic synapse	1.56 × 10^−6^	FOS, GSK3B*, PRKCA*, PPP2R5E, PPP2R3A, PPP2R2C, CREB5, DRD1, GRIA1, PPP3CC, CALM2*, GRIA2, PPP3CA, MAPK8, PPP2R2A, SCN1A, PPP2R3C, GNAQ, PPP2CB, AKT3*, CAMK2B, PPP1CB, GRIA3

* Denotes mRNA experimentally validated to interact with an miRNA from Table 2, as identified by TarBase v 7.0

## Discussion

Persistent pain is a common and poorly understood sequela of traumatic/stressful events such as MVC [46]. The results of this study show that even in the relatively small study sample, circulating blood miRNA in the first hours after stress exposure differed significantly among AAs who did and did not have severe persistent MVC-related AP 6 weeks later. Study results also provide an example of the novel pathophysiologic insights that may be obtained, as enrichment of X chromosome miRNA among differentially expressed miRNA, and sex differences in the effect of these miRNA, suggest potential mechanisms contributing to sex differences in vulnerability to persistent post-MVC pain. Supporting the role of these differentially expressed miRNA in persistent pain pathogenesis, these miRNA included a number of miRNA previously associated with the stress response and/or pain processing. However, further studies are needed to determine whether these miRNA play a causal role in the pathogenesis of pain post-MVC. Of note, study findings also support the legitimacy of persistent post-MVC musculoskeletal pain as a “real” disease outcome. This is important, as patients with post-MVC pain outcomes are highly stigmatized [47]. In addition, study findings also contribute to a growing literature indicating that stress-mediated changes in neurosensory processing play an important role in the pathogenesis of post-MVC outcomes.

We do not know whether miRNA predicting severe AP in the present study play a causal role in the development of these outcomes, or are markers for other cellular processes directly involved. If these miRNA play a causal role, mechanisms by which miRNA detected in blood may directly influence AP outcomes include: (1) miRNA expressed outside the central nervous system (CNS) and detected in the blood may cross the blood brain barrier [48, 49] to influence CNS processes, (2) miRNA expressed in the CNS may alter CNS transcription and also be released into the periphery (e.g., as part of a cellular/systemic communication system [50, 51]), (3) miRNA expressed outside the CNS may influence extra-CNS processes involved in the pathogenesis of post-traumatic pain [52, 53] (animal model data suggest that systemic, extra-CNS processes may play an important role in the pathogenesis of stress-induced pain [54]). Future studies are needed to better understand the identified associations between miRNA circulating in the immediate aftermath of trauma and the development of persistent pain states. Such studies may provide new insights into the biology of chronic pain development.

A potential causal role of the miRNA identified in the present study in the pathogenesis of severe post-MVC axial pain is supported by the known role of several of these miRNA in pain and/or stress-related processes. For example, miR-135a-5p binds the mineralcorticoid receptor (*NR3C2*) [55], the serotonin transporter (*SLC6A4*) [56], and the serotonin receptor-1a (*HTR1A*) [56] transcripts, all of which can affect pain processing (e.g. [57–59]), and miR-135a-5p has been shown to be expressed in pain-relevant tissues including the amygdala [60], 5-HT neurons [56], spinal cord [61], and pre-frontal cortex [62]. miR-19b-3p has been shown to be stress responsive in both the amygdala [63] and blood leukocytes [64], and can regulate the adrenergic receptor β-1 (*ADRB1*) [63]. Other miRNA, such as miR-3613-3p, have not been studied extensively; target prediction algorithms (e.g., TargetScan v7.0) predict that miR-3613-3p modulates the expression of pain-associated genes including *GABRB3*, *GRIN3A*, *TRPV1*, *NPY1R*, and *SCN9A*. Further examples of miRNA identified in this study that have prior associations with pain include miR-185-5p, miR-7-5p, and miR-26b-3p [61, 62, 65]. Equally important, many of the miRNA identified in this study are not currently known to be associated with stress or pain-related outcomes, suggesting that investigations such as the present study have the potential to lead to the identification of novel miRNA mediators.

An unexpected finding in the present study was that differentially expressed miRNA were enriched for miRNA located on the X chromosome. This finding holds true even accounting for the relatively high abundance of X chromosome miRNA expressed in the blood (8 %) compared to miRNA from other chromosomes. These X chromosomal miRNA were consistently expressed at higher levels in women than in men and appeared to contribute to persistent severe AP in women but not in men. These X chromosome miRNA were predicted to target pain-relevant transcripts from KEGG pathways known to be associated with various pain phenotypes, such as long term potentiation, neurotrophin signaling, and dopaminergic signaling [43–45]. Pathophysiologic mechanisms by which these miRNA may contribute to severe post-MVC AP in women but not in men are currently unknown. Six of the eight X chromosome miRNA identified to be associated with persistent severe AP in this study are transcribed as part of the miR-532-502 cluster of miRNA (a cluster which includes the expression of miR-532, -188, -500, -362, -501, -500b, -660, and -502). Upon examination of DNase hypersensitivity regions upstream of this cluster and upstream of miR-20b-5p and let-7f-2-3p, we did not find any obvious binding regions for sex hormone responsive transcription factors (TFs). However, let-7f-2-3p has been shown experimentally to be induced by estradiol and is in a dosage sensitive region of the X chromosome [66]. In addition to sex hormone responsive TFs, another mechanism that may contribute to the observed sex differences in miRNA effect is X chromosome inactivation. This phenomenon is believed to influence the transcription of ~15 % of all X chromosome genes [67, 68], and the results of at least two other studies suggest that miRNA genes can escape X chromosome inactivation. One study found that X chromosome miRNA genes are over-expressed in the T cells of women with lupus [69], and another study found sex-biased miRNA expression in the neonatal brain [70]. Further studies are needed to assess for associations between X chromosome miRNA expression and pain outcomes in men and women experiencing MVC, and to evaluate potential physiologic mechanisms by which sex differences in the expression of these miRNA may occur.

Some limitations should be considered when interpreting the results of this study. First, the sample size of this initial proof-of-concept study was relatively small. Future studies with much larger samples and greater power are needed. These studies should also adjust more stringently for multiple comparisons to reduce the probability of Type I error. Second, we did not adjust for potential confounders such as participant age, sex, or BMI in our primary analyses. However, adjusting for these factors in exploratory analyses did not diminish our effect size estimates. Third, we were not able to adjust for the potential confounding influence of medications administered in the ED and miRNA expression. We were able to evaluate chronic medication use in our cohort: no individuals in the study were taking opioids (due to exclusion criteria), 4 % of individuals were taking acetaminophen, and 13 % were taking NSAID. Adjusting for pre-MVC acetaminophen or NSAID use had negligible effect on effect size estimates for the association between miR-135a-5p and miR-3613-3p and severe AP development. Similarly, we were also unable to adjust for any potential confounding effect due to pre-MVC chronic illnesses, as comprehensive past medical history data on study participants was not available. Data from the emergency department record, for which past medical history data is often incomplete, indicated that the most prevalent chronic illnesses in the cohort were hypertension (25 %) and asthma (13 %). Hypertension was not associated with the development of severe AP (p = 0.757), and adjusting for hypertension did not weaken the association between miR-135a-5p and miR-3613-3p and severe AP development. Similar results were obtained when assessing the potential effect of asthma. Fourth, miRNA expression differences between those who did and did not develop severe AP were evaluated in the ED, in the early aftermath of MVC, and we do not know how the expression of these miRNA changed over time. However, we found that even in the very early aftermath of MVC, miRNA expression differed in those who did and did not subsequently develop persistent pain. Fifth, our study population was limited to African Americans, an understudied group that has been shown to experience an increased burden of adverse pain outcomes after trauma [71–74]. The generalizability of our findings to other ethnic groups is unknown. Sixth, larger sex specific strata are needed to fully understand miRNA expression differences associated with persistent severe AP development in women vs. men. Finally, pathway and gene target identification analyses were based on predicted binding rather than actual binding in biologic assays. However, predicted binding has been shown to have high concordance with actual binding, and predicted binding has the advantage of providing an unbiased assessment across the entire genome [75].

## Conclusion

The results of this study show that two miRNA, miR-135a-5p and miR-3613-3p, predict persistent AP development after MVC. In addition, study results suggest that X chromosome miRNA contribute to persistent pain development after MVC stress exposure in women, and that such miRNA may contribute to sex differences in vulnerability to persistent pain after MVC. More broadly, the results of this study support the hypothesis that analyses of miRNA collected from blood in the early aftermath of trauma/stress exposure might provide new insights into mechanisms of persistent pain development. Further studies are needed in larger samples of individuals experiencing MVC, both to validate current findings and to provide greater power to discover associated miRNA. Additionally, further experiments are needed to show whether the miRNA identified in this study play a causal role in persistent pain pathogenesis. The results of such studies may provide an important new window into these yet enigmatic processes.

## Methods

### Study design and setting

This prospective longitudinal study enrolled African American individuals who presented within 24 h of MVC to one of eight EDs in three states (Michigan, Pennsylvania, and Florida) between July 2012 and July 2013. The study only enrolled African Americans because of the pressing need for pain studies that focus on such understudied, high risk groups [74, 76–79]. The study was approved by the institutional review boards of all participating hospitals. Each participant provided written informed consent before enrollment.

### Participant eligibility criteria

Individuals ≥18 and ≤65 years of age presenting to the ED within 24 h of MVC who did not have fracture or other injury requiring hospital admission were screened for eligibility. Patients who were not alert and oriented were excluded, as were patients who did not self-identify as African American, pregnant patients, prisoners, patients unable to read and understand English, or patients taking opioids above a total daily dose of 30 mg of oral morphine or equivalent.

### Study procedures

Eligible and consenting participants provided a blood sample in the ED and completed an ED interview evaluation. Interview evaluations were performed by research assistants at the time of the ED visit using a web-based survey with explicit definitions of variables. Before enrolling patients in the ED, each research assistant completed a study training module followed by an interview with a standardized mock ED patient. Comparison of mock ED patient data across research assistants demonstrated an error rate of 0.57 %. Injury characteristics and medications administered in the ED were obtained by data extraction from the ED medical record. Six weeks after the MVC, participants completed a follow-up interview by telephone, online, or via mail. Participants were compensated $75 for completing the ED protocol and $50 for completing the 6-week interview.

### Participant demographics

Participant demographic characteristics (including age, gender, income, height, weight, and educational attainment) were obtained from the ED medical record and from participant self-report.

### Pain assessments and outcome definitions

Severity of pain in each body region during the month prior to MVC was assessed at the time of ED evaluation using a 0–-10 Numeric Rating Scale (NRS) score [80]. Severity of pain due to MVC in each body region was assessed at the 6 week time point using this same method together with an assessment of the MVC-relatedness of the pain. Individuals reporting a pain severity score ≥7 in at least one axial body region (neck, upper back, lower back, left shoulder, right shoulder) were defined as having severe AP [81, 82]. Individuals reporting severe AP during the month prior to the MVC were excluded from analyses.

### RNA collection and isolation

Research assistants collected blood samples in the ED at the time of enrollment using PAXgene RNA tubes. Total RNA (including miRNA) was isolated using the PAXgene blood miRNA kit (Qiagen, Valencia, CA, USA) and stored at −80 °C until use. RNA concentration and purity were measured using a NanoDrop 1000 (Nanodrop Technologies, Wilmington, DE).

### Library preparation and miRNA sequencing

Template libraries for miRNA Sequencing were produced from 1.0 ug total RNA using an adaptation of published protocols [83]. Briefly, total RNA was sequentially ligated to a 3′ linker using T4 RNA ligase 2, polyacrylamide gel extracted to remove excess 3′ linker, then ligated via T4 RNA ligase 1 to an oligonucleotide adapter (sequences shown in Additional file 4: Table S4). The 5′ adapter contained a two nucleotide barcode for multiplexing libraries. RNA products were reverse transcribed and amplified by PCR. In order to purify the miRNA population, gel isolation was used to obtain template libraries with 15–40 nucleotide inserts. Twelve barcoded libraries were combined per lane and sequenced on a HiSeq 2000 (Illumina, San Diego, CA, USA).

### Bioinformatics analysis and data normalization

Raw sequence reads were processed using a custom bioinformatics pipeline. Reads were de-multiplexed and barcode and adapter sequences removed. Mature miRNA sequences were obtained from miRbase v18.0 and genomic extensions were added before aligning with sequencing reads. Total read counts were generated including isomir and non-templated nucleotide addition. Sequencing reads were normalized using quantile normalization. In order to avoid individual samples with lowly abundant or no miRNA expression, miRNA species with less than 300 reads across samples were dropped from analyses (adapted as described previously [28]).

### RT-qPCR validation

The miRNA RT-qPCR method used is based on the stem-loop method described by Chen et al. [36]. Stem-loop RT primers and TaqMan probes for each miRNA were obtained from Life Technologies (Carlsbad, CA, USA). MiRNA expression of each miRNA was normalized to RNU48 levels before determination of expression differences. RT-qPCR validation was performed on a subset of the significant miRNA identified by miRNA sequencing (due to limited quantities of participant RNA).

### Statistical analysis

Differences in ED miRNA expression between those who did and did not subsequently develop AP were quantified by dividing the mean expression levels in the two groups. Because miRNA distributions were non-normal [Kolmogorov–Smirnov (K-S) test], expression levels of individual miRNA among individuals with and without AP, including for women and men subgroup analyses, were compared using the Wilcoxon-Mann–Whitney (WMW) test. Logistic regression models were used to test for sex-miRNA interactions while adjusting for age and ED study site. To account for multiple testing, we used the methods of False Discovery Rate (FDR) determination defined by Benjamini and Hochberg [35]. For this initial proof-of-concept discovery cohort, *p* value thresholds were set at p <0.05 and an FDR cut-off corresponding to 0.15. Statistical analyses were carried out using SPSS software version 21.0 or SAS University Edition.

### Identification of biologic pathways targeted by differentially expressed miRNA

A web-based computational tool, DIANA miRPath v2.0, was used to identify molecular pathways overrepresented in predicted targeting by differentially expressed miRNA originating from the X chromosome [79]. Pathway enrichment was also performed for the full set of 32 miRNA identified in Table 2. DIANA miRPath uses its predictive binding algorithm, DIANA-microT-CDS, to define a list of potential targets for each miRNA, then assigns a Kyoto Encyclopedia of Genes and Genomes (KEGG) pathway [41] rank and significance level based on the relative number of targets in that pathway [79]. DIANA miRPath results have been validated and its predictive binding algorithm has been shown to have high concordance with actual miRNA binding (e.g. [88, 90]). MiRNA that have been experimentally validated to bind to the predicted mRNA were identified using TarBase v2.0 [84]. The false discovery rates of the most highly ranked pathways were evaluated using permutation testing [42].

## Additional files

10.1186/s12990-015-0069-3 microRNA in whole blood collected from African Americans in the early aftermath of Motor Vehicle Collision (MVC) that are predictive of Axial Pain vs. Recovery 6 weeks after MVC trauma.
10.1186/s12990-015-0069-3 microRNA in whole blood collected from African American individuals in the early aftermath of Motor Vehicle Collision (MVC) that are predictive of Axial Pain vs. Recovery 6 weeks after MVC trauma, as assessed independently in women and men.
10.1186/s12990-015-0069-3 Top 10 DIANA miRPath predicted KEGG pathways enriched in targeting by all 32 miRNA differentially regulated in the early aftermath of MVC trauma in AA individuals who develop AP following MVC vs. those who recover.
10.1186/s12990-015-0069-3 Oligonucleotide sequences of linker adapters used in library preparation for miRNA sequencing.

## Abbreviations

MVC: motor vehicle collision
miRNA: microRNA
AP: axial pain
RT-qPCR: reverse transcription-quantitative polymerase chain reaction
ED: emergency department
AA: African American
KEGG: Kyoto Encyclopedia of Genes and Genomes
TF: transcription factor
NRS: numeric rating scale
FDR: false discovery rate
