# Supplementary material for: MicroRNA circulating in the early aftermath of motor vehicle collision predict persistent pain development and suggest a role for microRNA in sex-specific pain differences
Source: Mol Pain. 2015 Oct 24;11:66. doi: 10.1186/s12990-015-0069-3 (PMC4619556; doi:10.1186/s12990-015-0069-3)
Supplement: Supplementary file 1 — 10.1186/s12990-015-0069-3 microRNA in whole blood collected from African Americans in the early aftermath of Motor Vehicle Collision (MVC) that are predictive of Axial Pain vs. Recovery 6 weeks after MVC trauma. [file 12990_2015_69_MOESM1_ESM.docx]

| **Supplementary Table 1.** microRNA in whole blood collected from African Americans in the early aftermath of Motor Vehicle Collision (MVC) that are predictive of Axial Pain vs Recovery 6 weeks after MVC trauma | | | | | | | |
| --- | --- | --- | --- | --- | --- | --- | --- |
| **microRNA** | **Chr (Strand)^a^** | **Mean Fold Difference^b^** | **Avg seq reads (AP/noAP)** | **RT-qPCR Fold Chg** | **p value** | **Adjusted p value^c^** | **Interaction**  **p value (sex*miRNA)^d^** |
| miR-135a-5p | 3 (-) | 2.49 | 3.36/1.35 | 2.80 | **3x10^-4^** | **0.041** | 0.188 |
| miR-3613-3p | 13 (-) | 2.02 | 61.8/30.6 | 1.21 | **0.001** | **0.013** | 0.873 |
| miR-19b-3p | 13 (+) | 1.67 | 4201/2511 | 1.69 | 0.004 | **0.015** | 0.257 |
| miR-502-3p | X (+) | 1.75 | 258/147 | 3.79 | 0.004 | **0.003** | 0.400 |
| miR-500a-3p | X (+) | 1.39 | 53.0/38.1 |  | 0.005 |  | 0.251 |
| miR-1296-5p | 10 (-) | 1.99 | 8.80/4.43 |  | 0.006 |  | **0.017** |
| miR-454-5p | 17 (-) | 1.58 | 77.3/48.7 |  | 0.01 |  | 0.675 |
| miR-99a-5p | 21 (+) | 1.48 | 75.5/50.9 |  | 0.01 |  | 0.521 |
| miR-501-5p | X (+) | -1.15 | 52.4/60.4 |  | 0.011 |  | **0.071** |
| miR-362-5p | X (+) | 1.41 | 281/200 |  | 0.013 |  | **0.030** |
| miR-154-5p | 14 (+) | 1.09 | 4.01/3.68 |  | 0.015 |  | 0.226 |
| Let-7a-3p | 9 (+) | 1.48 | 49.8/33.8 |  | 0.02 |  | **0.022** |
| miR-185-5p | 22 (+) | -3.45 | 11019/38103 | -1.33 | 0.021 |  | 0.865 |
| miR-339-5p | 7 (-) | 1.31 | 544/415 |  | 0.023 |  | 0.590 |
| miR-29c-5p | 1 (-) | 1.67 | 156/93 |  | 0.023 |  | 0.161 |
| miR-4659b-3p | 8 (-) | -2.19 | 1.12/2.45 |  | 0.023 |  | 0.474 |
| miR-15b-5p | 3 (+) | -1.22 | 2536/3104 |  | 0.026 |  | 0.345 |
| miR-329-3p | 14 (+) | 1.68 | 6.94/4.12 |  | 0.026 |  | 0.512 |
| miR-20b-5p | X (-) | 1.35 | 328/243 |  | 0.029 |  | 0.878 |
| miR-500b-5p | X (+) | 1.38 | 10.56/7.63 |  | 0.029 |  | **0.054** |
| Let-7f-2-3p | X (-) | 1.43 | 2.35/1.64 |  | 0.029 |  | **0.015** |
| miR-7-5p | 9 (-) | -2.12 | 121/256 | -1.46 | 0.033 |  | 0.557 |
| miR-378a | 5 (+) | 1.37 | 199/145 |  | 0.034 |  | 0.671 |
| miR-3130-5p | 2 (-) | 1.91 | 11.27/5.89 |  | 0.034 |  | 0.927 |
| miR-532-5p | X (+) | 1.31 | 200/153 |  | 0.036 |  | 0.294 |
| miR-345-5p | 14 (+) | 1.62 | 145/90 |  | 0.037 |  | 0.871 |
| miR-16-5p | 13 (-) | -2.70 | 75003/202157 |  | 0.043 |  | 0.712 |
| miR-18a-3p | 13 (+) | 1.49 | 1138/766 |  | 0.044 |  | 0.540 |
| miR-337-3p | 14 (+) | -1.06 | 3.79/4.01 |  | 0.045 |  | 0.477 |
| miR-26b-3p | 2 (+) | -3.71 | 421/1562 | -1.78 | 0.046 |  | 0.201 |
| miR-26a-5p | 3 (+) | -2.52 | 3682/9262 |  | 0.048 |  | 0.739 |
| miR-151b | 8 (-) | 1.33 | 3846/2888 |  | 0.048 |  | 0.727 |
| ^a’^Chr’ refers to the chromosome name/number from where each miRNA is transcribed and ‘strand’ refers to whether it comes from the sense (+) or antisense (-) strand of the genome. ^b^Mean fold difference was calculated by dividing the average sequencing read counts for individuals developing AP by the average sequencing read counts for individuals who recover (as shown in the next column to the right). ^c^Linear regression models assessing miRNA association with WK6 pain scores from the region of the axial region with the most pain, adjusted for sex, age, and BMI. ^d^P value for the interaction term (sex*miRNA) in logistic regression analyses. Bolded p values are significant at p < 0.10 | | | | | | | |
