# Supplementary material for: MicroRNA circulating in the early aftermath of motor vehicle collision predict persistent pain development and suggest a role for microRNA in sex-specific pain differences
Source: Mol Pain. 2015 Oct 24;11:66. doi: 10.1186/s12990-015-0069-3 (PMC4619556; doi:10.1186/s12990-015-0069-3)
Supplement: Supplementary file 2 — 10.1186/s12990-015-0069-3 microRNA in whole blood collected from African American individuals in the early aftermath of Motor Vehicle Collision (MVC) that are predictive of Axial Pain vs. Recovery 6 weeks after MVC trauma, as assessed independently in women and men. [file 12990_2015_69_MOESM2_ESM.docx]

| **Supplementary Table 2.** microRNA in whole blood collected from African American individuals in the early aftermath of Motor Vehicle Collision (MVC) that are predictive of Axial Pain vs Recovery 6 weeks after MVC trauma, as assessed independently in women and men | | | | | | |
| --- | --- | --- | --- | --- | --- | --- |
| **microRNA** | **Chr (Strand)** | **Women Mean Fold Difference** | **p value, Women** | **Men**  **Mean Fold Difference** | **p value, Men** |  |
| mir-1296-5p | 10 (-) | 3.56 | **<0.001** | -1.15 | 0.847 |  |
| mir-502-3p | X (+) | 2.17 | **0.001** | 1.17 | 0.562 |  |
| let-7a-1-3p | 9 (+) | 2.81 | **0.001** | -1.34 | 0.949 |  |
| mir-135a-1-5p | 3 (-) | 3.32 | **0.001** | 1.92 | 0.088 |  |
| let-7f-2-3p | X (-) | 6.98 | **0.001** | -2.06 | 0.797 |  |
| mir-362-3p | X (+) | 3.54 | **0.002** | -2.42 | 0.088 |  |
| mir-3613-3p | 13 (-) | 1.95 | **0.003** | 2.12 | 0.171 |  |
| let-7b-3p | 22 (+) | 1.96 | **0.004** | -1.41 | 0.652 |  |
| mir-339-5p | 7 (-) | 1.65 | **0.006** | -1.06 | 0.699 |  |
| mir-548d-1-3p | 8 (-) | 2.72 | **0.006** | -1.42 | 0.401 |  |
| mir-454-5p | 17 (-) | 1.86 | **0.008** | 1.28 | 0.365 |  |
| mir-4326-5p | 20 (+) | 2.98 | **0.008** | -1.77 | 0.243 |  |
| mir-3130-1-5p | 2 (-) | 1.98 | **0.008** | 1.80 | 0.949 |  |
| mir-19b-1-3p | 13 (+) | 2.08 | **0.009** | 1.39 | 0.193 |  |
| mir-500a-3p | X (+) | 1.39 | **0.009** | 1.37 | 0.300 |  |
| mir-193a-3p | 17 (+) | 2.49 | **0.009** | 1.16 | 0.898 |  |
| mir-16-1-5p | 13 (-) | -4.17 | **0.012** | -1.56 | 0.898 |  |
| mir-20b-5p | X (-) | 1.47 | **0.012** | 1.12 | 0.847 |  |
| mir-29c-5p | 1 (-) | 1.94 | **0.012** | 1.39 | 0.438 |  |
| let-7f-1-3p | 9 (+) | 2.34 | **0.012** | 1.09 | 0.949 |  |
| mir-874-3p | 5 (-) | 1.12 | **0.012** | -1.54 | 0.606 |  |
| mir-500b-5p | X (+) | 2.59 | **0.014** | -1.36 | 0.606 |  |
| mir-26b-3p | 2 (+) | -5.37 | **0.015** | -2.38 | 0.652 |  |
| mir-4677-3p | 1 (+) | 3.43 | **0.015** | -2.70 | 0.101 |  |
| mir-584-5p | 5 (-) | 1.43 | **0.019** | -1.19 | 0.699 |  |
| mir-769-5p | 19 (+) | 1.38 | **0.019** | 1.00 | 0.898 |  |
| mir-1287-5p | 10 (-) | 3.77 | **0.019** | 1.08 | 0.898 |  |
| mir-30b-5p | 8 (-) | 1.66 | **0.021** | 1.23 | 0.847 |  |
| mir-15b-5p | 3 (+) | 1.07 | **0.021** | -1.71 | 0.365 |  |
| mir-4646-3p | 6 (-) | 1.58 | **0.021** | -1.99 | 0.401 |  |
| mir-501-3p | X (+) | 1.44 | **0.024** | -1.20 | 0.365 |  |
| mir-28-5p | 3 (+) | 1.68 | **0.024** | 1.18 | 0.898 |  |
| mir-409-3p | 14 (+) | 1.14 | **0.024** | 1.01 | 0.898 |  |
| mir-548e-3p | 10 (+) | 2.21 | **0.024** | -1.87 | 0.606 |  |
| mir-192-5p | 11 (-) | 1.84 | **0.027** | -1.37 | 0.699 |  |
| mir-3200-5p | 22 (+) | -4.36 | **0.027** | -1.53 | 0.699 |  |
| mir-494-3p | 14 (+) | 1.68 | **0.027** | -1.20 | 0.562 |  |
| mir-616-5p | 12 (-) | 1.60 | **0.027** | -1.13 | 0.699 |  |
| mir-1271-5p | 5 (+) | 2.18 | **0.027** | 1.14 | 0.898 |  |
| mir-491-5p | 9 (+) | 1.48 | **0.03** | 1.03 | 0.847 |  |
| mir-378a | 5 (+) | 1.52 | **0.033** | 1.17 | 0.478 |  |
| mir-548o-2-3p | 20 (+) | 1.96 | **0.033** | -1.27 | 0.365 |  |
| mir-570-3p | 3 (+) | 2.47 | **0.033** | -3.08 | 0.562 |  |
| mir-501-5p | X (+) | -1.41 | **0.037** | 1.46 | 0.217 |  |
| mir-1306-3p | 22 (+) | -1.81 | **0.037** | -2.59 | 0.949 |  |
| mir-671-3p | 7 (+) | 1.65 | **0.041** | -1.60 | 0.438 |  |
| mir-26a-1-5p | 3 (+) | -3.73 | **0.045** | -1.59 | 0.606 |  |
| mir-106b-3p | 7 (-) | 1.44 | **0.045** | -1.47 | 0.606 |  |
| mir-29b-1-3p | 7 (-) | 1.78 | **0.045** | -1.06 | 0.562 |  |
| mir-660-5p | X (+) | 1.91 | **0.045** | -1.24 | 0.606 |  |
| mir-589-5p | 7 (-) | 1.33 | **0.045** | -1.45 | 1.000 |  |
| mir-625-5p | 14 (+) | 1.56 | **0.045** | 1.21 | 0.652 |  |
| mir-190a-5p | 15(+) | 2.12 | **0.045** | -1.15 | 0.332 |  |
| mir-129-1-3p | 7 (+) | 1.54 | **0.045** | -1.46 | 0.652 |  |
| mir-362-5p | X (+) | 1.32 | **0.049** | 1.56 | 0.151 |  |
| mir-7-1-5p | 9 (-) | -2.34 | **0.049** | -1.92 | 0.332 |  |
| mir-671-5p | 7 (+) | 2.06 | **0.049** | -1.19 | 1.000 |  |
| miR-181b-1-5p | 1 (-) | 1.34 | 0.711 | -2.23 | **0.028** |  |
| mir-96-5p | 7 (-) | 1.10 | 0.654 | -3.79 | **0.019** |  |
| mir-188-5p | X (+) | 1.02 | 0.599 | -2.88 | **0.04** |  |
| mir-142-5p | 17 (-) | 1.63 | 0.338 | -3.07 | **0.04** |  |
| mir-1343-3p | 11 (+) | 1.22 | 0.202 | -3.46 | **0.034** |  |
| mir-99a-5p | 21 (+) | 1.36 | 0.072 | 1.71 | **0.047** |  |
|  | | | | | | |

All 32 miRNA and miRNA in women only
