# Supplementary material for: MicroRNA circulating in the early aftermath of motor vehicle collision predict persistent pain development and suggest a role for microRNA in sex-specific pain differences
Source: Mol Pain. 2015 Oct 24;11:66. doi: 10.1186/s12990-015-0069-3 (PMC4619556; doi:10.1186/s12990-015-0069-3)
Supplement: Supplementary file 3 — 10.1186/s12990-015-0069-3 Top 10 DIANA miRPath predicted KEGG pathways enriched in targeting by all 32 miRNA differentially regulated in the early aftermath of MVC trauma in AA individuals who develop AP following MVC vs. those who recover. [file 12990_2015_69_MOESM3_ESM.docx]

| **Supplementary Table 3.** Top 10 DIANA miRPath predicted KEGG pathways enriched in targeting by all 32 miRNA differentially regulated in the early aftermath of MVC trauma in AA individuals who develop AP following MVC versus those who recover. | |
| --- | --- |
| **Kegg Pathway** | **P value** |
| Pathways in cancer | 1.0x10^-54^ |
| MAPK signaling | 3.4x10^-41^ |
| PI3K-Akt signaling | 6.7x10^-40^ |
| Wnt signaling | 2.4x10^-34^ |
| Ubiquitin mediated proteolysis | 3.5x10^-30^ |
| Regulation of actin cytoskeleton | 6.2x10^-30^ |
| HTLV-I infection | 6.2x10^-30^ |
| Insulin signaling | 2.3x10^-24^ |
| Focal adhesion | 1.2x10^-23^ |
| Neurotrophin signaling | 2.0x10^-23^ |
|  | |
