# Supplementary material for: MicroRNA circulating in the early aftermath of motor vehicle collision predict persistent pain development and suggest a role for microRNA in sex-specific pain differences
Source: Mol Pain. 2015 Oct 24;11:66. doi: 10.1186/s12990-015-0069-3 (PMC4619556; doi:10.1186/s12990-015-0069-3)
Supplement: Supplementary file 4 — 10.1186/s12990-015-0069-3 Oligonucleotide sequences of linker adapters used in library preparation for miRNA sequencing. [file 12990_2015_69_MOESM4_ESM.docx]

| **Supplementary Table 4.** Oligonucleotide sequences of linker adapters used in library preparation for miRNA sequencing | |
| --- | --- |
| 5' Adapter AC barcode | GUUCAGAGUUCUACAGUCCGACGAUCACC |
| 5' Adapter AU barcode | GUUCAGAGUUCUACAGUCCGACGAUCAUC |
| 5' Adapter AG barcode | GUUCAGAGUUCUACAGUCCGACGAUCAGC |
| 5' Adapter AA barcode | GUUCAGAGUUCUACAGUCCGACGAUCAAC |
| 5' Adapter GA barcode | GUUCAGAGUUCUACAGUCCGACGAUCGAC |
| 5' Adapter GU barcode | GUUCAGAGUUCUACAGUCCGACGAUCGUC |
| 5' Adapter CA barcode | GUUCAGAGUUCUACAGUCCGACGAUCCAC |
| 5' Adapter CU barcode | GUUCAGAGUUCUACAGUCCGACGAUCCUC |
| 5' Adapter UU barcode | GUUCAGAGUUCUACAGUCCGACGAUCUUC |
| 5' Adapter UA barcode | GUUCAGAGUUCUACAGUCCGACGAUCUAC |
| 5' Adapter UC barcode | GUUCAGAGUUCUACAGUCCGACGAUCUCC |
| 5' Adapter UG barcode | GUUCAGAGUUCUACAGUCCGACGAUCUGC |
| 3' Adapter | P-UCGUAUGCCGUCUUCUGCUUGUddT |
